# The Usefulness of Admission Plasma NT-pro BNP Level to Predict Left Ventricular Aneurysm Formation after Acute ST-Segment Elevation Myocardial Infarction

**DOI:** 10.5935/abc.20190226

**Published:** 2019-12

**Authors:** Savas Celebi, Ozlem Ozcan Celebi, Serkan Cetin, Hande Ozcan Cetin, Mujgan Tek, Serkan Gokaslan, Basri Amasyali, Berkten Berkalp, Erdem Diker, Sinan Aydogdu

**Affiliations:** 1 TOBB Ekonomi ve Teknoloji Universitesi - Cardiology, Ankara - Turkey; 2 University of Health Science, Turkiye Yuksek Ihtisas Training and Research Hospital - Cardiology, Ankara - Turkey; 3 Afyon Kocatepe Universitesi Tip Fakultesi, Afyon - Turkey

**Keywords:** Myocardial Infarction, Coronary Aneurysm/complications, Myocardial Revascularization, Indicators of Morbidity and Mortality, Stroke Volume

## Abstract

**Background:**

Left ventricular aneurysm (LVA) is an important complication of acute myocardial infarction. In this study, we investigated the role of N- Terminal pro B type natriuretic peptide level to predict the LVA development after acute ST-segment elevation myocardial infarction (STEMI).

**Methods:**

We prospectively enrolled 1519 consecutive patients with STEMI. Patients were divided into two groups according to LVA development within the six months after index myocardial infarction. Patients with or without LVAs were examined to determine if a significant relationship existed between the baseline N- Terminal pro B type natriuretic peptide values and clinical characteristics. A p-value < 0.05 was considered statistically significant.

**Results:**

LVA was detected in 157 patients (10.3%). The baseline N- Terminal pro- B type natriuretic peptide level was significantly higher in patients who developed LVA after acute MI (523.5 ± 231.1 pg/mL vs. 192.3 ± 176.6 pg/mL, respectively, p < 0.001). Independent predictors of LVA formation after acute myocardial infarction was age > 65 y, smoking, Killip class > 2, previous coronary artery bypass graft, post-myocardial infarction heart failure, left ventricular ejection fraction < 50%, failure of reperfusion, no-reflow phenomenon, peak troponin I and CK-MB and NT-pro BNP > 400 pg/mL at admission.

**Conclusions:**

Our findings indicate that plasma N- Terminal pro B type natriuretic peptide level at admission among other variables provides valuable predictive information regarding the development of LVA after acute STEMI.

## Introduction

Left ventricular aneurysm (LVA) is an important prognostic marker that is strongly correlated with mortality and morbidity after acute ST-segment elevation myocardial infarction (STEMI). LVA is also strongly related to adverse clinical outcomes. It is well known that LVA carries a high risk of arrhythmia, thromboembolism and heart failure. Additionally, patients with this complication have a high risk of death within 1 year, independent of left ventricular ejection fraction.^1,2^

The factors that are associated with LVA after acute STEMI have already been determined. However, most of these studies were performed before the modern treatment era for myocardial infarction. Additionally, the biochemical predictors of this complication have not yet been determined. Early detection prior to the development of LVA may be helpful in the management of patients with acute STEMI.

N terminal pro-B-type natriuretic peptide (NT-pro BNP) is a 32-amino acid peptide that is synthesized and released predominantly from the ventricular myocardium in response to myocyte stretching.^3^ However, NT-pro BNP is secreted not only in response to increased left ventricular wall stretch but also to myocardial ischemia and infarction. Levels of NT-pro BNP correlate with left ventricular dilatation, remodeling, and dysfunction in patients after acute myocardial infarction.^4^

NT-pro BNP concentrations increase rapidly over the first 24 hours after acute myocardial infarction and then tend to stabilize. When measured 1 to 7 days after acute myocardial infarction, NT-pro BNP elevation identifies patients at risk for left ventricular dysfunction, heart failure, and death.^5-8^ NT-pro BNP levels after acute myocardial infarction have proven useful for predicting prognosis and estimating infarct size, but the value of NT-pro BNP for the prediction of LVA formation has not yet been determined.

The aim of this study was to evaluate the value of admission NT-pro BNP level in predicting LVA after acute STEMI.

## Methods

A total of 1,519 consecutive acute STEMI patients admitted to our department were enrolled in this study from June 2011 to January 2017. The protocol for the study was approved by the local ethics committee. The study complied with the Declaration of Helsinki guidelines. Written informed consent was obtained from all patients. The eligibility criteria included patients aged 21 to 75 years who presented within 12 h of chest pain. The exclusion criteria included previous heart failure, shock, pulmonary edema requiring intubation, and creatinine clearance < 30 ml/min. Acute STEMI was defined according to the third universal definition of myocardial infarction.^9^

Demographic information was collected, and a physical examination was performed for each patient. A 16-lead electrocardiogram recording was obtained from each patient immediately after admission.

Two-dimensional transthoracic echocardiography (TTE) was performed in all patients at admission and at the end of the first and six months of the index acute STEMI. The TTE measurements were performed using a Vivid 7 system (Vivid 7, GE Vingmed Ultrasound, Horten, Norway). The echocardiographic assessment was performed according to a previous study by Weyman et al.^10^ Complete 2-dimensional TTE, including Doppler flow interrogation, was performed according to standard techniques. LVA was defined as a demarcated bulge of the contour of the left ventricular wall during both diastole and systole, which showed akinesia and dyskinesia.

Blood samples were obtained immediately after admission to the coronary care unit using EDTA-containing tubes. The samples were stored for 3 days prior to NT-pro BNP assessment. Plasma NT-pro BNP level was measured using the Roche Diagnostics ElecsysproBNPelectrochemiluminescence immunoassay (ElecsysproBNP; Roche Diagnostics, Indianapolis, Ind). Baseline serum creatinine clearance was estimated using the Cockcroft-Gault formula. Fasting blood samples were taken in the morning after admission to determine fasting glucose and blood lipids. Blood samples for troponin I and creatine kinase-MB (CK-MB) assessment were taken every 8 h during the first 3 days after admission. The peak troponin and CK-MB levels during the hospital stay were also collected.

Reperfusion was achieved with primary percutaneous coronary intervention (PPCI) or fibrinolytic therapy. The choice of reperfusion therapy type was made according to the patient’s condition and the center’s capabilities. Patients who were not suitable for reperfusion therapy because of late admission, comorbidities or contra-indications were followed medically.

All patients underwent coronary angiography except patients with serious comorbidities or contra-indications. Selective left and right coronary angiography was performed using the Judkins technique. Left ventriculography was performed in the 30º right anterior oblique and 60° left anterior oblique projections and left ventricular end-diastolic pressure was measured before ventriculography.

The Rentrop grading scale was used to quantify the extent of collateral filling. The most opacified projection was used for grading. The following values were assigned according to the scale: 0 = no visible filling of any collateral vessel or collateral channels, 1 = filling of side branches of the artery to be perfused by collateral vessels without visualization of the epicardial segment, 2 = partial filling of the epicardial artery by collateral vessels, or 3 = complete filling of the epicardial artery by collateral vessels.

The mean collateral score was then calculated by dividing the sum of the Rentrop numbers by the number of patients.

The Gensini score was used to evaluate coronary lesion severity. Gensini score calculation was initiated by giving a severity score to each coronary stenosis as follows: 1 point for ≤ 25% narrowing, 2 points for 26 to 50% narrowing, 4 points for 51 to 75% narrowing, 8 points for 76 to 90% narrowing, 16 points for 91 to 99% narrowing, and 32 points for total occlusion. Thereafter, each lesion score was multiplied by a factor that considered the importance of the lesion's position in the coronary circulation (5 for the left main coronary artery; 2.5 for the proximal segment of the left anterior descending coronary artery; 2.5 for the proximal segment of the circumflex artery; 1.5 for the mid-segment of the left anterior descending coronary artery; 1.0 for the right coronary artery, the distal segment of the left anterior descending coronary artery, the posterolateral artery, or the obtuse marginal artery; and 0.5 for the other segments). Finally, the Gensini score was calculated by the summation of the individual coronary segment scores in each group.

PPCI performed only for the culprit artery. Percutaneous coronary intervention (PCI) was performed for non-culprit stenotic lesions during index hospitalization. Patients who received fibrinolytic therapy and subsequently underwent coronary angiography, ad hoc PCI was performed in patients with suitable coronary anatomy, and drug-eluting stents were used in most of the patients. In patients without suitable coronary anatomy for PCI, medical therapy or coronary artery bypass graft were decided.

All patients received aspirin (300-500 mg), a loading dose of clopidogrel (300-600 mg), and a bolus of unfractionated heparin (60-100 U/kg). At discharge, medical therapy was prescribed according to the patient’s individual status and guideline recommendations for secondary prevention.^11^

The patients were divided into two groups according to the presence of LVA within the six months of index MI. Group 1 consisted of patients with LVA, and group 2 included those without LVA.

### Statistical analysis

Statistical analysis was performed using IBM SPSS Statistics 22.0 statistical software. Continuous variables with normal distribution were reported as the mean ± standard deviation, continuous variables with non-normal distribution were reported as median -interquartile range and categorical variables were expressed as the number of patients and percentages. Normality was tested using the Kolmogorov-Smirnov test. Comparisons between categorical variables were performed by the Pearson’s chi-square test, or Fisher’s exact test, as appropriate. Continuous variables were compared by Student’s t-test for independent samples or the Mann-Whitney test, as appropriate. All tests were two-tailed, and a p-value < 0.05 was considered statistically significant. We analyzed the effects of different variables on the occurrence of ventricular aneurysm in univariate analysis and determined the variables whose unadjusted p-value was < 0.10 as potential risk markers and these were included in the full model. We composed the model by using forward elimination at multivariate regression analysis, and we eliminated potential risk markers by using likelihood ratio tests.

Area under the ROC (receiver operating characteristic) curves, based on C-statistics, were performed to determine the optimal cut-off value for NT-pro BNP to predict the LVA.

## Results

**Figure 1:**
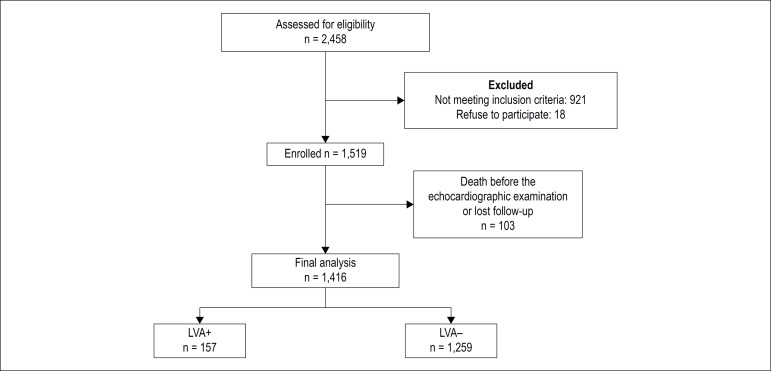
Patients flow chart demonstrating the number of patients eligible for inclusion into the study.

**Figure 2:**
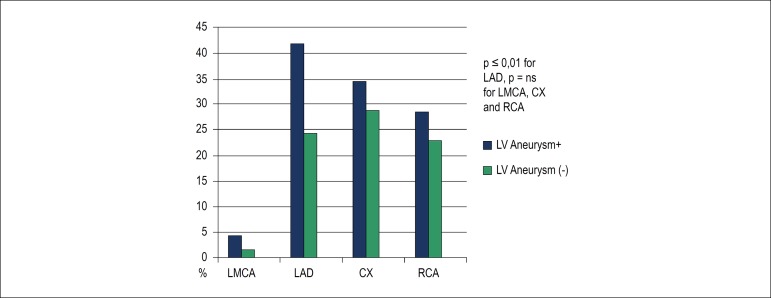
Culprit Artery in Patients with Left ventricular aneurysm. LV: left ventricle; LMCA: left main coronary artery; LAD: left anterior descending coronary artery; CX: circumflex artery; RCA: right coronary artery.

**Figure 3:**
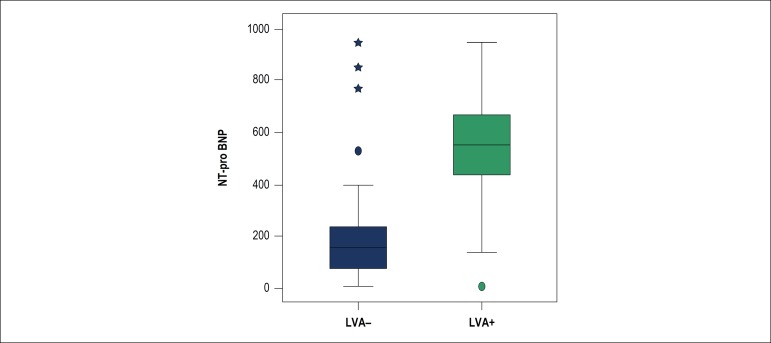
Patients who developed left ventricular aneurysm after acute ST-elevation myocardial infarction had higher NT-pro BNP levels at admission. NT-pro BNP: N terminal pro-B-type natriuretic peptide LVA: left ventricular aneurysm.

A total of 1,519 patients were enrolled (mean age 56.7 ± 11.7 years). Figure 1 describes the enrollment of patients for this study. The baseline characteristics of the patients are summarized in Table 1. The time from onset of chest pain to arrival at the hospital was 6 ± 12 hours. Of the 1,519 patients, primary PCI was performed in 67%, and 26% received fibrinolytic therapy. Seven percent of patients did not receive any reperfusion therapy. Among the patients who developed LVA, the myocardial infarction localization was anterior wall in 90.4%, isolated inferior wall in 1.3%, inferior-posterior in 2%, and inferior-right ventricle in 6.3%. In addition, anterior-located STEMI patients developed LVA more frequently than did patients with infarction on the other side (7.8% vs 1.2% p < 0,01). Patients who developed LVA had lower reperfusion rate than did patients without LVA (42.1% vs. 15.2%, p = 0.021) (Table 2). The LVA rate was lower in patients who received PPCI than in patients who received fibrinolytic therapy or no reperfusion therapy (5.3 vs. 9.2 p < 0.01, 5.3 vs. 14.7; p = 0.03). The type of fibrinolytic agent used had no effect on LVA development. Patients with LVA had a lower rate of P2Y12 inhibitor use (Table 3). When the culprit artery was the left anterior descending artery, the LVA risk was more than in the other coronary arteries (Figure 2). The Gensini scores were similar between groups (38.7 ± 30.8 vs. 37.9 ± 29.9, p = 0.924). However, Rentrop scores were significantly higher in patients without LVA (1.96 ±1.32 vs. 1.51 ± 0.76, p = 0.001). Time to reperfusion therapy was shorter in patients without LVA (4.1 ± 6.3 vs 6.2 ± 5.9, p < 0.05). The basal NT-pro BNP level was significantly higher in patients with LVA (523.5 ± 231.1 pg/mL vs. 192.3 ± 176.6 pg/mL, p < 0.001) (Figure 3). Multivariate logistic regression analysis determined the predictors of LVA after MI (Table 4). Previous CABG, post-MI heart failure, younger age, smoking, no-reflow phenomenon and high NT-pro BNP at admission predicted LVA formation after acute STEMI.

**Table 1:** Demographics of study group

	LV Aneurysm(-)	LV Aneurysm (+)	p-value
Age (years)	55.4 ± 11.0	61.0 ± 13.2	0.048
Gender (M) (n)%	(783)62.2	(106)67.5	0.070
BMI	27.4 ± 4.7	28.6 ± 2.9	0.030
Smoking (n) %	(673)53.4	(35)22.2	0.011
DM (n) %	(319)25.3	(36)22.9	0.803
Previous CABG (n) %	(23)1.8	(14)8.9	0.008
Previous PCI (n) %	(34)2.7	(2)1.3	0.439

LV: left ventricle; BMI: body mass index; DM: diabetes mellitus; CABG: coronary artery bypass greft; PCI: percutaneous coronary intervention.

**Table 2:** Laboratory and angiographic parameters of the study group

	LV Aneurysm (-)	LV Aneurysm (+)	p- value
NT-BNP, pg/mL	192.3 ± 176.6	523.5 ± 231.1	0.000
Gensini Score	38.7 ± 30.8	37.9 ± 29.9	0.924
Rentrop Score	1.96 ± 1.32	1.51 ± 0.76	0.000
Revascularization, %	72	45.5	0.021
Succesful Reperfusion, %	84.5	62.8	0.01
Killip	1.1 ± 0.3	1.5 ± 0.5	0.001
LVEF (%)	4.2 ± 8.6	32.3 ± 4.8	0.001
C r e atinine, mg/dl	0.9 ± 0.7	1.3 ± 0.8	0.02
MPV, fL	8.6 ± 0.8	9.3 ± 0.8	0.000
MCV, fL	85.0 ± 9.8	86.2 ± 10.7	0.683
Glucose, mg/dl	98.7 (IQR 62.0-312.0)	101.5 (IQR 66.0-427.0)	0.211
HbA1c, %	7.2 (IQR 5.8-13.1)	7.8 (IQR 6.5-14.8)	0.098
Peak cTnI, ng/mL	28.6 ± 19.2	43.4 ± 26.8	0.000
Peak CK-MB, IU/L	82.0 ± 54.30	212 ± 96.80	0.003

LVEF: Left ventricular ejection fraction; MPV: Mean platelet volume; MCV: mean corpuscular volume; IQR: Interquartile range; cTnI: cardiac troponine I; CK-MB: creatinine kinase-MB. Categorical variables were compared by Pearson's chi-square test or Fisher's exact test, and continuous numerical variables were compared by the Student's t test for independent sample or Mann-Whitney test, as appropriate.

**Table 3:** In hospital Therapy and Adverse Events

	LV Aneurysm(-) (n = 1259) (%)	LV Aneurysm(+) (n = 157) (%)	p-value
P2Y12 inh.	(1226) 97.3	(112) 71.3	0.008
LMWH	(912)72.4	(97)61.7	0.008
Statin	(124)98.6	(142)90.4	0.128
b-bloker	(1208)96.0	(136)86.6	0.300
ACE inh	(1041)82.6	(128)81.5	0.927
ARB	(34)2.7	(9)5.7	0.075
Spironolakton	(92)7.3	(22)14.0	0.128
Furosemide	(167)13.2	(99)63.1	< 0.0001
Tiyazid	(27)2.1	(14)8.9	0.050
CCB	(59)4.7	(7)4.4	0.542
Amiodorone	(67)5.3	(16)10.2	0.190
Digoxin	(16)1.3	(21)13.3	0.035
Warfarin	(75)5.9	(15)9.5	0.050
Insülin	(101)8.0	(16)10.1	1.000
OAD	(185)14.6	(7)4.4	0.205
Post MI angina	(34)2.7	(8)5.0	0.542
Heart Failure	(185)14.7	(93)59.2	0.01
Acut Renal Failure	(44)3.3	(7)4.4	0.404
Pericarditis	(51)4.0	(9)5.7	1.000
Arrythmia	(251)20.0	(64)40.7	0.046
GIS bleeding	(21)1.7	(4)2.5	1.000
Hematuri	(14)1.1	(7)4.4	0.227
LV thrombus	(23)1.8	(29)18.4	0.002
Mitral Regurgitation	(167)13.2	57(36.3)	0.015

P2Y12 inh.: P2Y12 inhibitor; LMWH: Low molecular weight heparine; ACE inh.: angiotensin converting enzyme inhibitor; ARB: Angiotensin receptor blocker; CCB: Calcium channel Blockers; OAD: Oral antidiabetic; MI: myocardial infarction; GIS: Gasrointestinal system; LV: left Ventricle. Categorical variables were compared by Pearson's chi-square test or Fisher's exact test.

**Table 4:** Effects of variables on left ventricular aneurysm formation after acute MI in univariate and multivariate logistic regression analysis

Varible	Univariate logistic regression analysis			Multivariate logistic regression analysis		
	OR	95% CI	p	OR	95% CI	p
Age (> 65y)	1.04	1.00 - 1.09	0.051	1.04	1.00 - 1.09	0.030
BMI	1.06	0.96 - 1.17	0.263			
NT-proBNP (> 400 pg/mL)	1.01	1.00 - 1.01	0.005	1.01	1.00 - 1.01	0.001
Gensini Score	1.00	0.98 - 1.02	0.923			
Killip Class (> 2)	8.96	2.90 - 28.96	0.0001	9.71	3.10 - 30.43	0.007
Peak cTnI [ng/mL]	2.86	1.49 - 5.50	0.002	3.02	1.86 - 4.97	0.0 60
LVEF(< 50%) after MI	0.82	0.75 - 0.90	0.051	0.85	0.82 - 0.96	0.070
Peak CK-MB (IU/L)	0.36	0.01 - 0.65	0.005	0.23	0.12 - 1.02	0.001
Smoking	0.32	0.05 - 0.82	0.010	0.26	0.09 - 0.77	0.015
HT	1.81	0.65 - 5.01	0.253			
DM	0.87	0.28 - 2.67	0.803			
**Medication**						
Statin	0.14	0.01 - 1.57	0.109			
B Blocker	0.40	0.06 - 2.54	0.329			
ACE Inh.	0.94	0.27 - 3.25	0.927			
ARB	5.76	0.90 -	0.165			
Spironolactone	7.40	0.64 - 85.82	0.192			
Furosemide	5.94	1.0 - 36.33	0.180			
Fibrinolytic therapy	1.74	0.15 - 20.12	0.058	1.88	0.30 - 23.8	0.620
Post MI HF	9.10	2.97 - 21.12	0.003	8.40	2.90 - 24.35	0.001
ARF	3.52	0.21 - 58.76	0.080	3.65	0.28 - 56.32	0.100
Post MI Pericarditis	1.14	0.11 - 11.57	0.910			
Arrhythmia	2.77	1.00 - 7.69	0.510			
Previous CABG	11.37	3.81 - 33.98	0.001	4.29	1.19 - 15.50	0.026
Failure of reperfusion	0.34	0.20 - 1.46	0.050	0.32	0.12 - 0.86	0.024
No-reflow phenomenon	0.98	0.48 - 1.33	0.025	0.96	0.42 - 1.22	0.012

BMI: body mass index; cTnI: cardiac troponin I; LVEF: left ventricular ejection fraction; MI: Myocardial Infarction HT: Hypertension, DM: Diabetes Mellitus; CE: Angiotensin converting enzym inhibitor; ARB: angiotensin receptor blocker; Post MI HF: Post Myocardial infarction heart failure; ARF: acute renal failure; CABG: Coronary artery by-pass greft.

**Figure 4:**
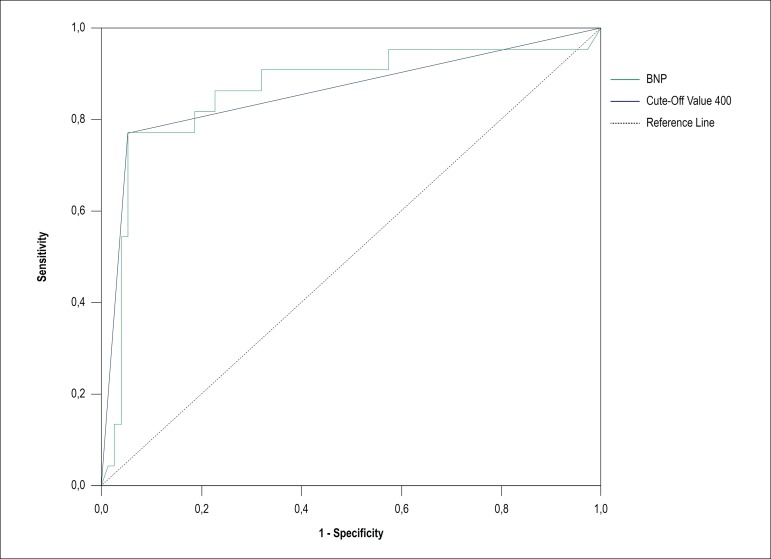
Receiver Operating Characteristic (ROC) Curve analysis shows the cut-off value of NT-pro BNP to predict left ventricular aneurysm.

Roc analysis showed that the cut-off value of NT-pro BNP at admission for LVA development was 400 pg/mL. The sensitivity and specificity were 78.3% and 94.7%, respectively. (Area under curve: 0.860 0.751-0.968 95% CI) (Figure 4).

Roc analysis showed that the cut-off value for peak cTnI for LVA development was 78 pg/mL (Area under the curve: 0.720 0.541-1.320 95% CI) and for peak CK-MB was 312.86 IU/mL (Area under the curve: 0.640 0.314-0.986 95% CI).

## Discussion

Our study showed two main issues: 1) the factors affecting the development of LVA in the new treatment era of acute STEMI, and 2) that NT-pro BNP measured during the acute phase of STEMI is useful for predicting LVA development.

Recent studies have determined that LVA incidence after acute STEMI was reduced from 10-30% to 8-15% after the developments in the treatment of acute STEMI.^12,13^ In accordance with these data, we found that the incidence of LVA after acute STEMI was 10.3%. Although there are some controversial issues, previous studies have determined that single-vessel disease, total LAD occlusion, poor collateral supply to the infarct-related artery, hypertension and female gender were major determinants for the development of LVA after acute MI.^14-16^ However, most of these data were reported before the modern treatment era. In our study, we determined that patients who received fibrinolytic therapy or no reperfusion therapy developed LVA more frequently than did patients who received PPCI. The type of fibrinolytic agent used had no effect on LVA formation. Patients who received reperfusion therapy earlier had less frequent LVA formation. These data showed that earlier reperfusion prevents the development of LVA. Moreover, in our study, P2Y12 inhibitors were found to be a determinant of LVA formations. Hirai et al.^17^ showed that good collateral coronary circulation has a beneficial effect on the prevention of LVA formation.^17^ Similarly, we found that the Rentrop score was significantly higher in patients without LVA. We also determined that the severity of coronary disease had no effect on the development of LVA. Additionally, gender and risk factors such as diabetes or hypertension had no effect on the development of LVA.

The biomarkers of cardiac function may provide useful information in evaluating cardiac outcomes after acute STEMI. Mayr et al.^18^ found that NT-pro BNP on day 3 after admission correlated with acute and chronic infarct size and left ventricular ejection fraction after acute myocardial infarction.^18^ Kleczyński et al.^19^ showed that the assessment of NT-pro BNP level 6 months after STEMI is a useful marker of infarct size and left ventricle function at long-term follow up.^19^ Fazlınezhad et al.^20^ showed that BNP level is a predictor of acute MI complications such as left ventricular pseudoaneurysm.^20^ We showed that NT-pro BNP assessment within the first 12 hours of chest pain is a good indicator to predict LVA. This relationship may be associated with ischemic remodeling of the left ventricle, which triggers NT-pro BNP secretion.

The answer as to why we observed high NT-pro BNP levels before the development of LVA may be associated with the properties of NT-pro BNP. The level of NT-pro BNP may reflect the severity of the ischemic insult, even when myocardial necrosis has not occurred. It has been shown that in experimental acute myocardial infarction, NT-pro BNP synthesis is augmented not only in infarcted tissue but also in non-infarcted tissue.^21^ In addition, NT-pro BNP levels have been shown to increase transiently after uncomplicated percutaneous transluminal coronary angioplasty, even when intracardiac filling pressures remain unchanged.^22^ Therefore, we may suggest that transient ischemia increases wall stress and induces BNP synthesis and the release in proportion to the degree of ischemic insult. Afterwards, this ischemia causes infarct tissue and LVA. Thus, we observed high levels of NT-pro BNP before the development of LVA.

## Conclusion

In this study, we found that in the modern treatment era of acute STEMI, there are new factors such as reperfusion therapy or P2Y12 inhibitors that affect the development of LVA. We also found that NT-pro BNP > 400 pg/dL measured during the first 12 hours of acute STEMI is a good predictor of LVA formation. To the best of our knowledge, no previously published studies have demonstrated the relationship between admission NT-pro BNP levels and LVA formation after acute MI. Therefore, we concluded that a single measurement of NT-pro BNP at admission in patients with acute STEMI proves useful for the estimation of LVA development.
